# The Role of Generative Artificial Intelligence in Developing Cognitive and Research Talent Among Postgraduate Students

**DOI:** 10.3390/jintelligence14040053

**Published:** 2026-03-26

**Authors:** Asem Mohammed Ibrahim, Reem Ebraheem Saleh Alhomayani, Azhar Saleh Abdulhadi Al-Shamrani

**Affiliations:** Department of Education and Learning, College of Education, King Khalid University, Abha 61421, Saudi Arabia; ralhmyani@kku.edu.sa (R.E.S.A.); azhar@kku.edu.sa (A.S.A.A.-S.)

**Keywords:** generative artificial intelligence, cognitive and research talent, postgraduate students

## Abstract

Generative Artificial Intelligence (GAI) is rapidly transforming higher education by introducing new mechanisms for supporting the development of advanced cognitive processes and research-related capabilities. This study examines how postgraduate students employ GAI to develop their cognitive and research talent, conceptualized here as higher-order academic skills such as analysis, synthesis, and critical reasoning, across six domains: literature review, theoretical development, research design, data analysis, academic writing, ethical use, and challenges encountered—signaled explicitly rather than listed line by line. We administered a validated multidimensional scale to 214 postgraduate students, and the results indicate a moderate overall use of GAI, with notably high involvement in practices that emphasize ethics and responsibility. Students reported clear cognitive benefits in tasks involving information processing, linguistic refinement, and conceptual clarification while showing caution toward delegating higher-order analytical or theoretical reasoning to AI systems. Key challenges included limited institutional training, concerns about data privacy and academic integrity, and difficulties evaluating the originality and reliability of AI-generated content. Inferential analyses indicated significant differences based on gender, academic level, and general technology proficiency, whereas no differences emerged across age groups, departments, or specializations. Overall, this study demonstrates how GAI can contribute to the development of higher-level cognitive skills and research competencies, with “moderate use” operationalized as consistent but selective engagement across domains, while underscoring the need for structured training, clear guidelines, and teaching approaches that foster the responsible and effective incorporation of AI within postgraduate research. The results highlight practical implications for higher education, including the importance of institutional training programs, governance frameworks for responsible AI use, and pedagogical models that foster critical engagement with GAI.

## 1. Introduction

The rapid advancement of Generative Artificial Intelligence (GAI) is reshaping the foundations of higher education, research practices, and talent development. As universities worldwide integrate AI-driven tools into academic environments, a growing body of scholarship highlights the transformative potential of GAI in enhancing creativity, cognitive intelligence, and socio-emotional growth—dimensions that align closely with contemporary models of talent development in the digital era (Jabli et al., 2025; Alshamsi et al., 2024; Zawacki-Richter et al., 2019). Postgraduate education, in particular, stands at the center of this transformation, as students at advanced academic levels increasingly rely on AI-enabled systems to support complex research tasks, deepen cognitive engagement, and refine scholarly outputs (Suchanek & Kralova, 2025; Vieriu & Petrea, 2025).

In recent years, GAI has evolved from simple content-generation tools into more sophisticated cognitive partners capable of supporting analytical reasoning, conceptual development, and creative problem-solving. This shift has broadened its educational value beyond efficiency, positioning GAI as a potential contributor to higher-order thinking and research-oriented competencies. Empirical studies demonstrate that GAI enhances students’ ability to produce high-quality digital content, stimulates creative thinking, and supports innovation in academic contexts (Jabli et al., 2025; Alshamsi et al., 2024). It also facilitates big-data analysis, pattern recognition, and visualization, enabling postgraduate students to engage with complex research tasks that traditionally required advanced methodological expertise (Suchanek & Kralova, 2025; Vieriu & Petrea, 2025).

Despite this growing body of research, much of the existing research focuses on general educational contexts or undergraduate populations, resulting in an incomplete grasp of the ways postgraduate students employ GAI to cultivate the interconnected areas of cognitive ability and research skills. These domains represent essential capabilities for advanced academic work, yet they remain underexplored in relation to AI-supported learning environments.

In order to bridge this gap, the current research introduces a more precise definition of “cognitive and research talent”. Within the scope of this research, cognitive talent is defined as advanced capabilities including analytical reasoning, the ability to solve problems, metacognitive regulation, and conceptual integration—skills that recent scholarship associates with the cognitive influence of generative AI and its contribution to enhancing complex thought processes (Chen et al., 2025; Y. Zhao et al., 2025; Lee et al., 2025). Research talent, in turn, encompasses competencies such as methodological literacy, academic writing, evidence evaluation, and the synthesis of complex information, all of which have been shown to benefit from AI-supported research practices and AI-enabled analytical tools (Castillo-Segura et al., 2024; Sushereba et al., 2025). This refined definition provides a more coherent theoretical framework that guides the study’s objectives and the structure of its measurement approach.

Students’ engagement with GAI is shaped by factors such as perceived usefulness, ease of use, AI literacy, and alignment with broader educational goals (Arpaci et al., 2025; Long & Magerko, 2020; Zawacki-Richter et al., 2019). These factors may influence how postgraduate students integrate GAI into their research practices, potentially affecting the development of their cognitive and research abilities (Suchanek & Kralova, 2025; Yang, 2025). However, the extent to which GAI contributes to genuine talent development—rather than simply expediting the completion of academic tasks—remains insufficiently understood.

Global guidelines, including those issued by UNESCO, emphasize the importance of ethical and responsible use of GAI in education (UNESCO, 2023). These guidelines highlight the need for transparency, accountability, and human oversight in AI-assisted learning and research, echoing broader concerns in the literature on academic integrity and responsible AI use (Amini et al., 2025; Tlili et al., 2025). For postgraduate students, who must navigate ethical considerations while leveraging AI tools, these guidelines are particularly relevant.

Collectively, these developments highlight the importance of conducting a targeted inquiry into the ways postgraduate students engage with GAI to strengthen their cognitive and research capacities, as well as examining how this engagement differs across demographic and academic profiles.

## 2. Research Problem

The rapid integration of Generative Artificial Intelligence (GAI) into higher education has reshaped expectations for learners, educators, and institutions. As universities adopt AI-enabled systems to enhance learning, research, and talent development, postgraduate students stand at the center of this transformation (Alshamsi et al., 2024; Zawacki-Richter et al., 2019). Their academic journey—marked by advanced cognitive demands, rigorous research tasks, and the need for scholarly independence—positions them to benefit significantly from GAI’s capabilities (Suchanek & Kralova, 2025; Vieriu & Petrea, 2025).

Studies show that students’ perceptions of GAI vary widely depending on prior experience, cultural context, and exposure to AI-driven tools. Research conducted on Saudi university students reports generally positive attitudes toward GAI, with learners recognizing its potential to enhance academic performance and streamline complex tasks (Aldossary et al., 2024; Alshamsi et al., 2024). International studies report similar findings, where students view GAI as helpful for feedback, assessment, and learning efficiency (Kizilcec et al., 2024; J. Zhao et al., 2024). However, positive perceptions do not necessarily translate into meaningful or effective use—particularly when the goal is to cultivate higher-order cognitive abilities and research competencies (Chen et al., 2025; Qi et al., 2025). This gap between perception and actual utilization underscores the necessity of exploring how postgraduate students interact with GAI to advance cognitive growth, instead of limiting its use to simple task execution.

The broader educational landscape reflects a complex interplay of opportunities and challenges associated with GAI. Scholars highlight GAI’s potential to reduce cognitive load, improve access to knowledge, and support innovative learning strategies (Arpaci et al., 2025; Stein, 2025; Zawacki-Richter et al., 2019). Yet these same technologies are also shaped by concerns related to ethics, accuracy, algorithmic bias, and the danger of excessive dependence on AI-produced material (Amini et al., 2025; Dinçer, 2024; Miao et al., 2024; Ersöz & Engin, 2024). These concerns are particularly relevant for postgraduate students, who are expected to uphold high standards of academic integrity and methodological rigor.

Despite the promising literature on GAI in education, substantial shortcomings persist in understanding how postgraduate students specifically engage with GAI to enhance the full spectrum of cognitive and research talent. Most existing studies focus on undergraduate populations, general educational contexts, or isolated skills such as creativity or critical thinking (Y. Zhao et al., 2025; Qi et al., 2025). Few studies have examined the comprehensive role of GAI in supporting the intertwined domains of cognitive and research talent among postgraduate learners, who face unique academic demands and expectations.

Drawing upon these gaps, this research explores the degree to which postgraduate students utilize GAI to advance their cognitive and research capacities, while also analyzing how such utilization differs according to gender, age, academic department, program level, academic status, specialization, technological engagement, and intensity of interaction with GAI tools.

## 3. Research Questions

This investigation focused on the following research questions:

1. To what extent do postgraduate students employ Generative Artificial Intelligence (GAI) to foster Cognitive and Research Talent?
2. Do notable variations exist in postgraduate students’ utilization of GAI for enhancing Cognitive and Research Talent when considered across gender, age, academic department, program level, academic standing, specialization, degree of technology adoption, and intensity of GAI engagement?

## 4. Research Objectives

This study aimed to achieve the following objectives:

1. Investigate the ways in which postgraduate learners employ Generative Artificial Intelligence (GAI) to foster the growth of cognitive and research abilities.
2. Examine differences in the utilization of GAI among postgraduate students across demographic and academic variables.
3. Formulate evidence-driven suggestions aimed at strengthening the productive application of GAI in advancing cognitive and research skills within higher education.

## 5. Research Methodology

A descriptive quantitative design was adopted to examine how postgraduate learners make use of Generative Artificial Intelligence (GAI) in developing Cognitive and Research Talent. This methodological choice proved suitable, as it allowed for a structured analysis of usage patterns, degrees of engagement, and differences across demographic, academic, and technological dimensions. The quantitative framework supported the gathering and interpretation of numerical evidence to assess overall levels of use and to detect statistically meaningful distinctions among subgroups, thereby offering a robust means of tracing technology adoption trends in higher education. In this study, the descriptive quantitative methodology entailed the use of structured survey instruments to collect standardized responses, allowing for the computation of means, standard deviations, and percentages that summarize students’ perceptions and experiences. This design emphasizes describing the current state of GAI utilization rather than testing causal relationships, thereby providing a clear picture of prevailing practices and challenges. Importantly, the validated multidimensional scale employed in this study was explicitly aligned with the domains of cognitive and research talent outlined earlier, thereby reinforcing methodological coherence and ensuring consistency between the theoretical framing and the empirical design.

## 6. Research Sample

The research sample comprised 214 postgraduate learners from the College of Education at King Khalid University. Participation was entirely voluntary, allowing individuals to decide freely on their involvement. The sample encompassed a wide spectrum of academic departments, program levels, study stages, and specializations, thereby offering a balanced and representative view of the postgraduate community. For clarity, the profile can be outlined as follows: most were doctoral candidates (56.07%), nearly half reported strong engagement with technology (50.93%), and a considerable share were concentrated in Teaching and Learning (50.93%) as well as Educational Leadership and Policy (41.59%). This diversity strengthens the applicability of the study’s outcomes to comparable educational contexts. Table 1 provides an overview of the demographic and academic features of the 214 postgraduate participants included in the research.

As shown in Table 1 the demographic and academic profile of the sample reflects a well-balanced and diverse group of postgraduate students. Males represent 53.74% and females 46.26%. The age distribution shows a mature cohort, with 22% under 30 years old, 38.79% between 30–40, and 39.25% above 40. Academically, most students are enrolled in Teaching and Learning (50.93%) or Educational Leadership and Policy (41.59%), while Psychology accounts for 7.48%. Doctoral students form the majority (56.07%), and 29.44% are in the thesis preparation stage. Specializations vary, with Educational Administration and Supervision (32.24%) and Science Curriculum and Instruction (23.83%) being the most common. Technological engagement is high, as 50.93% report high technology use, and GAI use ranges from moderate (46.73%) to high (37.38%). Such diversity reinforces the rigor of the study and enhances the applicability of its results to comparable higher education settings.

## 7. Research Tool

For this investigation, data were gathered through a self-administered survey designed to evaluate the extent to which postgraduate learners engage with Generative Artificial Intelligence (GAI) in developing cognitive and research capacities. The tool utilized a ten-point rating scale, enabling respondents to indicate nuanced degrees of agreement and frequency of use, in line with earlier studies on AI-facilitated learning and advanced cognitive skills (Cheng et al., 2025; Qi et al., 2025; Y. Zhao et al., 2025). To preserve complete anonymity and confidentiality, no personal identifiers—such as names or email addresses—were collected.

The Scale of Generative Artificial Intelligence Use in Enhancing Research and Cognitive Talents (GAI-RCT Scale) comprised six dimensions reflecting the principal domains through which GAI may contribute to the advancement of cognitive and research talent among postgraduate students:

1. GAI-Supported Cognitive Skills in Literature Review and Theoretical Framework Development (GAI-LRTF)
2. GAI-Enhanced Research Design and Problem-Formulation Skills (GAI-RDPF)
3. GAI-Assisted Data Analysis and Interpretation Skills (GAI-DAI)
4. GAI-Driven Academic Writing and Research Refinement Skills (GAI-AWRR)
5. Ethical and Responsible Use of GAI in Research (GAI-ERU)
6. Challenges in Employing GAI for Cognitive and Research Development (GAI-CRD C)

The GAI-RCT Scale included 48 items distributed evenly across these dimensions. Its development was informed by an extensive review of relevant literature and previously validated instruments in the fields of artificial intelligence in education, cognitive engagement, and academic integrity (Amini et al., 2025; Long & Magerko, 2020; Tlili et al., 2025; Zawacki-Richter et al., 2019). In line with cognitive load and higher-order thinking perspectives, items were designed to capture both the intensity and depth of GAI use in research tasks (Sweller, 2020; Y. Zhao et al., 2025). This equal distribution was informed by the theoretical foundations established in prior literature, ensuring balance across domains and reinforcing the validity of the scale. Such coherence between the theoretical framing and the measurement structure enhances confidence in the reliability of the results. The GAI-RCT Scale was administered electronically, and participation was entirely voluntary. Participants were briefed on the objectives of the study beforehand and were granted the freedom to omit any question, thereby upholding ethical research principles and reducing the likelihood of response bias.

### 7.1. Psychometric Properties: Validity and Consistency

#### 7.1.1. Validity

Face validity was confirmed through an expert evaluation process. A panel comprising seven specialists in educational psychology, educational technology, and curriculum and pedagogy carefully reviewed the instrument. Their assessment focused on clarity, relevance, and cultural appropriateness, and their unanimous consensus verified that the questionnaire was appropriate for capturing the intended constructs among postgraduate learners.

#### 7.1.2. Internal Consistency

The internal consistency of the instrument was assessed following its administration to the complete sample of 214 postgraduate learners. Table 2 displays the Pearson correlation values linking each item to its respective dimension, along with the correlations between individual items and the total scale score, thereby offering empirical support for the reliability of the instrument.

The results in Table 2 show strong and statistically significant Pearson correlations between each item and its corresponding dimension, indicating a high level of internal coherence across the instrument. Most items correlate above 0.80 with their dimensions, demonstrating that they consistently measure the intended constructs and that each dimension is clearly defined. Correlations between items and the overall scale are also substantial, generally ranging from 0.75 to 0.86, confirming that the items contribute meaningfully to the broader construct. Although a few items show moderate correlations with the total scale, their strong alignment with their specific dimensions suggests they capture focused subdomains within the instrument—an expected pattern in multidimensional measures. While some alpha values exceeded 0.90, this does not indicate redundancy; rather, it reflects the deliberate and even distribution of 48 items across the six domains, which enhances balance and strengthens confidence in the scale’s validity and reliability. Taken together, the correlation pattern offers compelling confirmation of the instrument’s internal consistency and affirms its reliability in evaluating postgraduate learners’ engagement with Generative Artificial Intelligence for advancing cognitive and research capacities.

Table 3 displays the Pearson correlation matrix, highlighting the interconnections among the six dimensions of the GAI RCT Scale and their linkage to the overall scale score.

The correlation matrix in Table 3 shows strong and statistically significant relationships among the scale’s core dimensions, confirming a coherent and well-structured measurement model. The four skill-based dimensions—covering GAI-supported cognitive skills, research design, data analysis, and academic writing—exhibit particularly high intercorrelations (0.801–0.884), indicating that students who use GAI in one stage of the research process tend to apply it across other stages as well. The ethical and responsible use dimension shows moderate correlations with these skills, suggesting that ethical awareness is related to GAI engagement but remains conceptually distinct from operational research competencies. In contrast, the challenges dimension displays weaker correlations, reflecting its role as an external constraint rather than a core component of cognitive or research talent. All dimensions correlate significantly with the total scale (0.462–0.917), providing strong evidence of structural validity and confirming that the instrument effectively captures the multifaceted contributions of GAI to cognitive and research talent development. Importantly, these correlation patterns support the multidimensionality of the conceptual model, demonstrating that the scale measures several related but distinct constructs rather than collapsing into a single overarching factor. This reinforces the theoretical framing and strengthens confidence in the structural validity of the instrument.

#### 7.1.3. Confirmatory Factor Analysis

Confirmatory factor analysis was employed to evaluate the factorial validity of the GAI RCT Scale within its six-dimensional framework, with each latent construct measured by eight observed indicators (totaling 48 items). The analysis was carried out using AMOS 26 and Maximum Likelihood Estimation (MLE) on data from 214 postgraduate learners. All items demonstrated significant loadings on their designated latent dimensions, with standardized coefficients typically ranging from 0.72 to 0.85—well above the accepted threshold of 0.50—thereby affirming that the indicators appropriately capture their theoretical constructs. Residual variances were found to be low to moderate, offering additional evidence of the robustness and reliability of the measurement model.

The model fit indices revealed that the six-factor configuration achieved an acceptable level of fit: χ^2^ = 1922.6, df = 959, χ^2^/df = 2.00, CFI = 0.928, TLI = 0.915, IFI = 0.929, and RMSEA = 0.069 (90% CI = 0.064–0.073). Although the NFI value (0.867) fell slightly below the recommended benchmark, the collective pattern of indices affirmed the suitability of the proposed model.

Convergent validity was additionally substantiated, with Composite Reliability (CR) values surpassing 0.70 and Average Variance Extracted (AVE) values exceeding 0.50 across all six dimensions, thereby confirming that the scale demonstrates adequate convergent validity.

Table 4 presents the fit indices derived from the confirmatory factor analysis (CFA) conducted to validate the six-factor structure of the GAI RCT Scale. The table outlines the principal indices, their observed values, recommended thresholds, and interpretations, offering evidence of the model’s adequacy.

As shown in Table 4, The CFA results demonstrate that the six-factor model of the GAI-RCT Scale achieved an overall acceptable fit. The χ^2^/df ratio (2.00) falls within the recommended range, and incremental fit indices (CFI = 0.928, TLI = 0.915) exceed the 0.90 threshold, indicating strong model adequacy. Although the NFI (0.867) is slightly below the recommended cutoff and SRMR (0.086) marginally exceeds the threshold, the RMSEA (0.069) remains within acceptable limits. Taken together, these indices confirm that the hypothesized six-factor structure provides a satisfactory representation of the data, thereby supporting the construct validity of the instrument.

**Figure 1:**
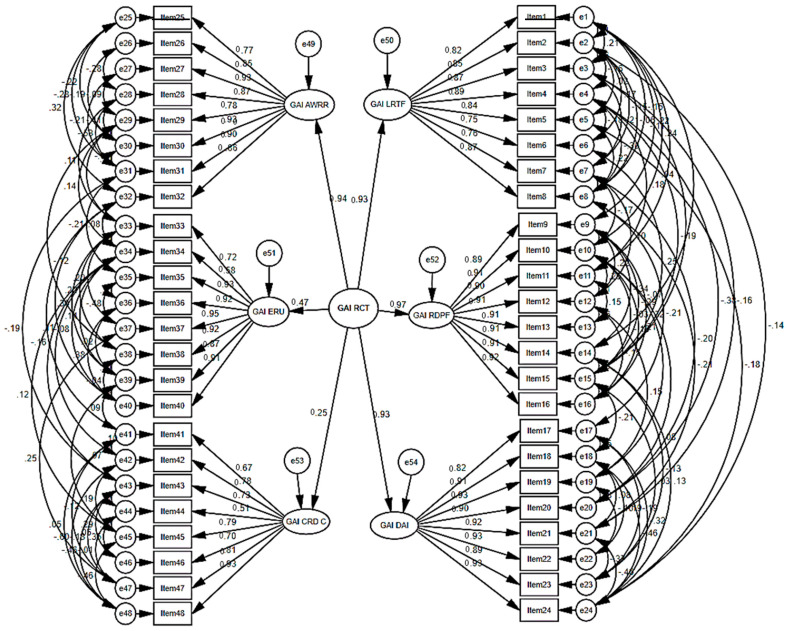
Unified CFA Model for the Six-Factor Model of the GAI-RCT Scale.

Figure 1 presents the confirmatory factor analysis (CFA) measurement model for the GAI-RCT Scale in its six-factor structure. Each latent construct is represented by eight observed items, with standardized regression weights linking items to their respective dimensions. The diagram also illustrates the higher-order factor (GAI-RCT) and its relationships with the six latent dimensions, alongside error variances for each item.

Figure 1 demonstrates that all items exhibited significant loadings on their designated latent dimensions, with standardized coefficients typically falling within the range of 0.67 to 0.85, exceeding the recommended threshold of 0.50. The higher-order factor (GAI-RCT) demonstrated strong associations with most dimensions (e.g., 0.94, 0.93, 0.97), while one dimension showed a weaker relationship (0.25), reflecting the selective contribution of certain domains. The overall pattern supports the factorial validity of the six-factor model, confirming that the measurement structure adequately represents the theoretical constructs of the scale.

#### 7.1.4. Reliability

The reliability of the instrument was assessed through Cronbach’s alpha and the Guttman split-half coefficient, offering a thorough evaluation of its internal consistency. Table 5 reports the reliability coefficients obtained.

The reliability analysis in Table 5 shows exceptionally strong internal consistency across all six dimensions. Cronbach’s alpha values range from 0.910 to 0.974, far exceeding accepted reliability standards and confirming that items within each dimension consistently capture the constructs they are designed to measure. Guttman split-half coefficients (0.906–0.959) further support the instrument’s stability. The total scale also demonstrates outstanding reliability, with alpha at 0.979 and split-half at 0.984. Overall, these results indicate that the instrument is psychometrically robust, internally coherent, and well suited for assessing postgraduate students’ use of GAI in developing cognitive and research talent.

## 8. Statistical Analysis

Descriptive statistical measures, including means and standard deviations, were employed to evaluate the overall extent of GAI utilization in fostering cognitive and research talent. Group differences were examined using independent samples *t*-tests for two-category variables and one-way ANOVA for variables with more than two categories, followed by LSD post hoc tests when significant differences appeared. Students’ responses on the 1–10 scale were classified into five levels—very low to very high—by dividing the nine-point range into intervals of 1.8. This interval width was chosen to ensure equal distribution across the five interpretive levels, thereby enhancing transparency and consistency in how performance levels were derived. Table 6 reports the corresponding mean and percentage ranges for each level of GAI use. Additionally, assumptions underlying the *t*-tests and ANOVAs (such as normality and homogeneity of variances) were checked and met, reinforcing the methodological transparency and robustness of the statistical analysis.

## 9. Research Results

1. First question answer: To what extent do postgraduate students employ Generative Artificial Intelligence (GAI) to foster Cognitive and Research Talent?

To respond to this inquiry, descriptive statistical analyses—including means, standard deviations, and percentages—were calculated from participants’ responses across all scale items. Table 7 presents the aggregate level of Generative Artificial Intelligence (GAI) utilization in enhancing cognitive and research talent, together with the corresponding performance levels for each of the six dimensions among postgraduate learners.

The descriptive results in Table 7 indicate that postgraduate students show a moderate overall level of engagement with Generative AI in developing their cognitive and research abilities, with a mean score of 6.18 (61.80%). Ethical and responsible use records the highest mean (7.80; 78.03%), reflecting strong awareness of transparency, accuracy, and responsible application when using AI in academic work. GAI-supported cognitive skills in literature review and theoretical framework development rank second (6.35; 63.53%), suggesting that students find AI particularly useful for navigating complex texts and synthesizing information.

Moderate engagement is also observed in research design, academic writing, and data analysis (5.43–5.82), indicating uneven integration across research stages. The lower score for AI-assisted data analysis (5.43; 54.31%) may reflect limited familiarity with advanced analytical tools or uncertainty about AI-generated interpretations. Challenges related to GAI use also appear at a moderate level (6.02; 60.17%), pointing to obstacles such as technical limitations and insufficient training.

Overall, the findings suggest that students are in a transitional phase of adoption, with strong ethical awareness but incomplete practical integration, highlighting the need for targeted training and institutional support. In summary, ethical and responsible use shows the highest engagement, while data analysis reflects the lowest, indicating that students prioritize responsible application of GAI but remain less confident in employing it for advanced analytical tasks.

Table 8 presents the descriptive statistical results for the items of the first dimension of the GAI-RCT Scale, namely GAI-supported cognitive skills in literature review and theoretical framework development.

The results in Table 8 indicate that postgraduate students use GAI at a moderate level to support cognitive skills in literature review and theoretical framework development, with an overall mean of 6.35 (63.53%). This suggests meaningful engagement with AI tools during the early stages of research, though usage is not uniformly high across all tasks.

The most frequent practice is using GAI to translate content from international studies (M = 6.81; 68.08%), highlighting its value in accessing global research beyond language barriers. Students also report strong use of AI for identifying key concepts (M = 6.55) and summarizing lengthy studies (M = 6.44), reflecting the role of GAI in managing extensive academic literature efficiently.

Moderate use appears in tasks requiring deeper analysis—such as organizing literature, interpreting theories, and comparing perspectives—with means between 6.22 and 6.38. This suggests that students rely on AI for clarification and synthesis but still prefer personal judgment for theoretical interpretation.

The lowest mean relates to using AI to design the initial structure of the theoretical framework chapter (M = 5.82; 58.22%), indicating caution in delegating higher-order conceptual tasks.

Overall, students use GAI confidently for information-processing tasks while remaining more reserved in areas requiring advanced theoretical reasoning, reflecting a balanced and thoughtful integration of AI into their research practices. In summary, students show higher engagement with GAI in translation and summarization tasks, while their use is more cautious in structuring theoretical frameworks, underscoring a preference for AI in information management rather than conceptual modeling.

Table 9 presents the descriptive statistical results for the items of the second dimension of the GAI-RCT Scale, namely GAI-enhanced research design and problem-formulation skills.

The results in Table 9 show that postgraduate students use GAI at a moderate level to support research design and problem formulation, with an overall mean of 5.82 (58.18%). This indicates that students are beginning to integrate AI tools into early research planning, though their use remains cautious and not yet fully embedded in methodological practice.

The highest-rated use of GAI involves improving the clarity and suitability of research instrument items (M = 6.14; 61.36%), reflecting confidence in AI’s ability to refine wording and enhance precision. Students also report moderate use of GAI for generating questionnaire and interview items and organizing instrument domains (M = 5.84–5.90), suggesting that AI is viewed as a helpful assistant in structuring data-collection tools.

Lower means appear in tasks requiring deeper conceptual reasoning—such as formulating objectives, developing hypotheses, and identifying variable relationships (M = 5.62–5.76). This pattern suggests hesitation to rely on AI for theoretical modeling or foundational research decisions.

Overall, the findings depict a balanced yet cautious adoption of GAI. Students are comfortable using AI for procedural and practical tasks but remain selective when engaging with conceptual and analytical components of research design. In summary, students show stronger engagement with GAI in refining and structuring research instruments, while their use is weaker in formulating objectives and hypotheses, underscoring a tendency to rely on AI for practical rather than conceptual aspects of research design.

Table 10 presents the descriptive statistical results for the items of the third dimension of the GAI-RCT Scale, namely GAI-assisted data analysis and interpretation skills.

The results in Table 10 show that postgraduate students use GAI at a moderate level for data analysis and interpretation, with an overall mean of 5.43 (54.31%). This indicates that AI tools are beginning to enter the analytical phase of research, though their use remains cautious and not yet fully integrated into students’ routines.

The highest-rated practice is using AI to analyze findings and compare them with prior studies (M = 5.70; 56.96%), reflecting recognition of AI’s value in synthesizing results and situating them within existing literature. Students also report moderate use of AI to explain statistical concepts (M = 5.67) and identify patterns in qualitative data (M = 5.58), suggesting that GAI supports clarification and early-stage coding.

Moderate engagement is also seen in drafting initial versions of results and discussion sections, suggesting tables and charts, and linking findings to hypotheses (M = 5.36–5.58). These uses indicate reliance on AI for preliminary structuring, followed by human refinement.

The lowest means relate to interpreting statistical outputs (M = 5.00) and analyzing qualitative interviews (M = 5.08), reflecting caution in delegating tasks requiring precision and methodological rigor.

Overall, the findings suggest that GAI is becoming a supportive tool for explanation, synthesis, and early drafting, while students maintain careful oversight in areas demanding accuracy and nuanced interpretation. In summary, students show stronger engagement with GAI in synthesizing findings and explaining statistical concepts, while their use is weakest in interpreting statistical outputs and qualitative interviews, underscoring reliance on AI for supportive rather than precision-driven analytical tasks.

Table 11 presents the descriptive statistical results for the items of the fourth dimension of the GAI-RCT Scale, namely GAI-driven academic writing and research refinement skills.

The results in Table 11 show that postgraduate students use GAI at a moderate level to support academic writing and research refinement, with an overall mean of 5.66 (56.55%). The highest-rated uses involve preparing abstracts in Arabic and English (M = 6.02) and rephrasing long sentences into clearer academic language (M = 6.00), indicating that students value GAI for improving clarity, readability, and bilingual communication.

Moderate engagement is also observed in generating recommendations, enhancing linguistic quality, and standardizing citation styles (M = 5.64–5.68), suggesting that students rely on GAI for technical and stylistic refinement. Lower scores appear in tasks requiring structural or conceptual development, such as improving coherence across thesis chapters (M = 5.32) and generating research titles (M = 5.40). These results imply that students prefer to retain control over the intellectual organization of their work.

Overall, GAI is emerging as a useful tool for enhancing clarity and presentation, while students continue to exercise judgment in higher-level conceptual tasks. In summary, students show stronger engagement with GAI in improving clarity, abstracts, and stylistic refinement, while their use is weaker in structuring coherence and generating titles, underscoring reliance on AI for linguistic and technical support rather than conceptual organization.

Table 12 presents the descriptive statistical results for the items of the fifth dimension of the GAI-RCT Scale, namely the ethical and responsible use of GAI in research.

The results in Table 12 show that learners consistently exhibit a high level of ethical and responsible use of Generative AI, with an overall mean of 7.80 (78.03%). This indicates that students engage with AI tools while maintaining strong awareness of academic integrity, institutional expectations, and the potential risks linked to AI-generated content. The highest-rated item reflects students’ recognition of potential errors or hallucinations in AI outputs (M = 8.25), highlighting a critical and cautious approach to evaluating AI-produced information. Students also show strong commitment to ensuring that AI-generated content aligns with academic integrity standards and to viewing AI as a supportive tool rather than a substitute for scholarly effort (M = 8.10).

High levels of diligence are evident in verifying compliance with academic regulations (M = 8.07) and checking the accuracy of AI-provided data (M = 8.02). The lowest mean, though still high, relates to citing original sources suggested by AI (M = 6.44), possibly reflecting uncertainty about citation practices. Overall, the findings portray a student population that uses AI thoughtfully, balancing efficiency with responsible oversight. In summary, students show the strongest engagement in critically evaluating AI outputs and ensuring academic integrity, while relatively lower engagement appears in citation practices, underscoring a cautious yet responsible approach to integrating GAI into research.

Table 13 presents the descriptive statistics related to the challenges faced by postgraduate students in employing generative AI (GAI) for cognitive and research development.

The results in Table 13 indicate that postgraduate students face a moderate level of challenges when using generative AI for cognitive and research development, with an overall mean of 6.02 (60.17%). Although students are increasingly engaging with GAI tools, several obstacles still limit full integration into their research practices. The most significant challenge is the lack of adequate training within academic programs (M = 7.10), revealing a clear gap between rapid AI advancements and institutional support. Concerns about privacy and data protection also rank highly (M = 6.78), reflecting students’ awareness of the ethical and security risks associated with AI platforms.

Plagiarism-related concerns (M = 6.63) further highlight students’ caution regarding academic integrity. Moderate challenges appear in handling technical issues, assessing originality, and aligning AI-generated content with academic requirements (M = 5.63–6.11). The lowest means relate to distinguishing accurate from unreliable outputs (M = 5.22) and limited knowledge of effective AI use (M = 5.01).

In summary, the findings reveal that although students acknowledge the significance of GAI, practical, ethical, and technical barriers persist, underscoring the need for structured training, institutional guidance, and enhanced digital literacy. The synthesis of the results highlights that the most critical challenges are institutional in nature, particularly the lack of adequate training and support, followed by ethical concerns such as privacy and plagiarism. Technical and knowledge-related barriers appear at a moderate level. Importantly, the relatively large mean differences across items point to substantive challenges that carry practical significance, indicating the need for targeted interventions and structured guidance rather than being viewed as merely statistical variations.

2. Second question answer: Do notable variations exist in postgraduate students’ utilization of GAI for enhancing Cognitive and Research Talent when considered across gender, age, academic department, program level, academic standing, specialization, degree of technology adoption, and intensity of GAI engagement?

Independent samples *t*-tests were conducted to examine differences in postgraduate students’ use of generative AI across gender, program level, and technology engagement. Table 14 presents the results, including both statistical significance and effect sizes to provide a more comprehensive interpretation of the findings.

The results in Table 14 show several notable differences in postgraduate learners’ use of GAI to enhance cognitive and research talent. A statistically significant gender difference emerged (t = 2.417, *p* = 0.017), with male students reporting higher overall use of GAI (M = 312.25) than female students (M = 278.46). The effect size (d = 0.33) is moderate, suggesting that while the difference is meaningful, it is not large. This highlights the importance of pedagogical strategies that ensure equitable support for both male and female students in adopting AI-enhanced research practices.

No significant difference was found between master’s and doctoral students (t = −1.711, *p* = 0.088). The effect size (d = 0.17) is small, indicating that program level alone does not substantially influence GAI use. This suggests that both groups engage with AI at comparable levels, and differences in adoption are better explained by other factors such as digital readiness.

The strongest difference relates to students’ general level of technology use. Those with high technological engagement reported significantly greater use of GAI (M = 337.88) compared to those with moderate engagement (M = 253.79), with a highly significant result (t = 6.518, *p* < 0.001). The effect size (d = 0.89) is large, underscoring digital proficiency as a critical enabler of effective AI adoption.

By integrating effect sizes and pedagogical interpretation, the results move beyond statistical significance to emphasize their educational relevance. Overall, the findings suggest that gender and technological confidence influence GAI use, whereas program level does not. These patterns highlight the need for targeted support for students with lower digital readiness to ensure equitable access to AI-enhanced research practices. A more comprehensive examination of these implications is offered in the Section 10.

Table 15 reports the ANOVA findings that explore variations in postgraduate learners’ use of Generative Artificial Intelligence (GAI) across multiple demographic and academic variables, including age, department, academic level, specialization, and overall GAI utilization.

The ANOVA results in Table 15 show a varied pattern in the factors influencing postgraduate learners’ use of GAI to enhance cognitive and research talent. No significant differences were found for age, academic department, or program specialization (*p* > 0.05), indicating that students across demographic and disciplinary groups engage with GAI at comparable levels. This suggests that GAI adoption is broadly distributed rather than concentrated within specific segments of the postgraduate population.

In contrast, two variables demonstrate strong and statistically significant effects. Academic level shows a notably high F value (F = 249.19, *p* < 0.001), indicating substantial increases in GAI use as students advance through their programs—likely due to greater research demands and growing familiarity with digital tools. In a similar vein, the extent of Generative Artificial Intelligence (GAI) utilization emerged as a significant predictor (F = 79.64, *p* < 0.001), emphasizing the importance of digital preparedness and prior experience in influencing students’ participation in AI-supported research practices.

Overall, the findings suggest that while demographic and disciplinary factors exert limited influence, academic progression and technological proficiency are key drivers of GAI adoption among postgraduate students.

Table 16 displays the outcomes of the post hoc (LSD) analyses, which investigated variations in postgraduate learners’ utilization of Generative Artificial Intelligence (GAI) to advance SERTPs across different academic levels and degrees of GAI use.

The post hoc analyses presented in Table 16 provide a deeper understanding of the significant differences related to academic level and GAI-use level. For academic level, the LSD results show consistent and statistically significant differences across all groups, with students in the thesis preparation stage reporting the highest use of GAI. The large mean gaps—such as the 241-point difference between thesis-stage students and first-level students—indicate a sharp increase in GAI engagement as students progress academically. This pattern suggests that advanced students rely more heavily on GAI for literature synthesis, methodological refinement, data interpretation, and academic writing, whereas early-stage students appear more cautious, likely due to limited research experience.

A similarly clear pattern emerges for levels of GAI use. High users report significantly greater engagement than both moderate and low users, and moderate users differ significantly from low users. The substantial mean differences, including the 202-point gap between high and low users, highlight the pivotal importance of digital literacy and prior exposure in shaping effective GAI integration.

Overall, the findings indicate that academic progression and AI proficiency are the strongest predictors of GAI adoption, while demographic and disciplinary factors exert minimal influence. These results emphasize the need for structured training and early exposure to AI tools to ensure equitable and effective use among postgraduate students.

## 10. Results Discussion

### 10.1. Discussion of the First Research Question: To What Extent Do Postgraduate Students Employ Generative Artificial Intelligence (GAI) to Foster Cognitive and Research Talent?

The findings provide a comprehensive view of how postgraduate students engage with Generative Artificial Intelligence (GAI) to enhance their cognitive and research capabilities. The overall moderate level of use across the six dimensions suggests that students are in a transitional phase of adoption—neither fully integrating GAI into their research practices nor disregarding its potential. This pattern aligns with broader developments in higher education, where GAI is increasingly recognized as a powerful tool but remains unevenly embedded due to differences in digital readiness, institutional support, and prior exposure (Aldossary et al., 2024; Arpaci et al., 2025; Maxwell et al., 2025; Zawacki-Richter et al., 2019; Alshamsi et al., 2024). The moderate engagement observed here indicates that students value GAI but are still learning how to incorporate it strategically into their research workflows (Zhong & Rosli, 2025; Suchanek & Kralova, 2025). These findings not only confirm prior reports of uneven GAI adoption in higher education, but also extend them by showing how postgraduate students selectively integrate AI depending on task complexity.

#### 10.1.1. GAI-Supported Cognitive Skills Through Literature Review and the Development of Theoretical Frameworks

Students report moderate use of GAI for literature review and the development of theoretical frameworks, relying on it mainly for cognitively supportive tasks such as summarizing articles, clarifying complex ideas, and navigating extensive bodies of literature. These practices mirror previous findings showing that GAI can reduce cognitive load, enhance information processing, and support higher-order thinking when used appropriately (Cheng et al., 2025; Qi et al., 2025; Sweller, 2020; Y. Zhao et al., 2025). In this context, GAI functions as a “cognitive amplifier,” helping students manage the volume and complexity of scholarly texts, a challenge that is particularly pronounced at the postgraduate level (Zawacki-Richter et al., 2019; Yang, 2025).

However, students show greater caution when using GAI for deeper conceptual tasks such as structuring theoretical frameworks or defining core constructs. This selective reliance aligns with research indicating that, while GAI can support idea generation, it often lacks the epistemic grounding required for producing rigorous theoretical models independently (Ding et al., 2025; Benvenuti et al., 2023). Similar studies show that students prefer to retain intellectual control over interpretive and theory-building processes, even when using AI for preliminary synthesis (Wang et al., 2025; Zhang & Han, 2025; Sushereba et al., 2025). Overall, the findings suggest that students view GAI as a complementary cognitive partner rather than a substitute for human reasoning, consistent with current policy recommendations emphasizing human oversight in tasks requiring deep understanding (European Commission et al., 2025; UNESCO, 2023).

#### 10.1.2. GAI-Enhanced Research Design and Problem-Formulation Skills

The results for this dimension also show moderate and selective use. Students commonly employ GAI to generate questionnaire items, interview questions, and preliminary instrument structures—tasks that benefit from AI’s ability to produce organized and coherent content quickly. These practices align with studies demonstrating GAI’s usefulness in supporting systematic research tasks and early-stage idea generation (Castillo-Segura et al., 2024; Xiao et al., 2025; Malloy & Gonzalez, 2024).

However, students are more hesitant to rely on GAI for formulating research problems, defining objectives, or constructing hypotheses. These tasks require disciplinary knowledge, methodological rigor, and conceptual coherence—areas where students appear to prefer human judgment. This pattern is consistent with findings showing that AI adoption decreases as task complexity and the need for higher-order reasoning increase (Arpaci et al., 2025). Scholars also caution against delegating core conceptual decisions to AI due to risks of superficial coherence or hidden biases (Andersen et al., 2025; Dinçer, 2024; Amini et al., 2025). Thus, students seem to adopt a pragmatic division of labor: using GAI for procedural tasks while retaining responsibility for theory-driven aspects of research design. This aligns with recent work emphasizing the pivotal role of AI literacy and conceptual understanding when AI use is epistemically appropriate (Long & Magerko, 2020; Umanets et al., 2024; Tlili et al., 2025).

#### 10.1.3. GAI-Assisted Data Analysis and Interpretation Skills

Students report moderate yet cautious use of GAI for data analysis and interpretation. They frequently rely on GAI to clarify statistical concepts, identify qualitative themes, and generate preliminary interpretations—patterns consistent with research showing that GAI can support sense-making and early-stage analysis (Xiao et al., 2025; Sushereba et al., 2025). AI tools can also enhance analytical reasoning by simplifying technical explanations and offering structured interpretations (Sobolenko et al., 2024; Malloy & Gonzalez, 2024).

However, the lowest scores in this dimension relate to interpreting statistical outputs and analyzing qualitative interview data. These concerns reflect well-documented limitations of GAI, including the risk of hallucinations, oversimplified interpretations, and methodological inaccuracies (Maxwell et al., 2025; Ding et al., 2025). Students’ caution mirrors broader academic concerns about the epistemic reliability of AI in high-stakes analytical tasks (European Commission et al., 2025; Dinçer, 2024). Overall, students appear to view GAI as a supportive tool rather than a replacement for human analytical expertise, consistent with the consensus that human judgment remains essential for ensuring validity and methodological rigor (Zawacki-Richter et al., 2019; Y. Zhao et al., 2025).

#### 10.1.4. GAI-Driven Academic Writing and Research Refinement Skills

Students show moderate engagement with GAI in academic writing, valuing its ability to improve clarity, enhance linguistic quality, and support bilingual communication. These findings align with research demonstrating GAI’s effectiveness in improving readability, coherence, and stylistic quality (Abdelmagid et al., 2025; Suchanek & Kralova, 2025). Students appear to use GAI as a writing assistant that helps refine drafts, correct language errors, and strengthen argumentation.

However, lower scores for tasks involving structural coherence, title generation, and conceptual framing indicate that students prefer to maintain control over the intellectual architecture of their work. This preference is consistent with studies showing that GAI can enhance surface-level writing but cannot reliably generate deeper conceptual logic (Baskara, 2024; Benvenuti et al., 2023). Students therefore tend to rely on GAI for micro-level refinements while retaining human oversight for macro-level decisions (Yang, 2025; Vieriu & Petrea, 2025). These findings reinforce the view that GAI is best used as a supportive tool within a human-AI collaborative model (Tlili et al., 2025; Amini et al., 2025).

#### 10.1.5. Ethical and Responsible Use of GAI in Research

One of the most notable findings is the high level of ethical and responsible GAI use among postgraduate students. This strong ethical orientation aligns with global concerns about hallucinations, inaccuracies, and risks to academic integrity (Giannakos et al., 2025; Miao et al., 2024; Ersöz & Engin, 2024). UNESCO’s guidelines emphasize transparency, verification, and human oversight (UNESCO, 2023), and the results suggest that students are internalizing these principles.

High scores in this dimension also reflect growing awareness of the need to critically evaluate AI outputs, verify sources, and avoid uncritical reliance on AI-generated text. This aligns with research showing that ethical awareness is becoming a core component of AI literacy (Long & Magerko, 2020; Tlili et al., 2025). Students’ trust in AI tools appears strongly shaped by their understanding of ethical risks and their ability to apply responsible use strategies (Shahzad et al., 2025). Overall, the findings indicate that students are developing a mature, reflective approach to AI use that balances innovation with academic integrity.

#### 10.1.6. Challenges in Employing GAI for Cognitive and Research Development

The final dimension highlights several barriers that may hinder full integration of GAI into research practices. The most prominent challenge is the lack of adequate training within academic programs—a concern echoed in studies showing that AI advancements have outpaced curricular adaptation (Mbah et al., 2025). Without structured training, students may struggle to use GAI effectively, particularly for advanced research tasks.

Other challenges include technical difficulties, evaluating AI-generated content, and uncertainty about integrating AI outputs with traditional academic requirements. These concerns reflect broader issues related to accuracy, reliability, and methodological rigor (Nedungadi et al., 2024; Nikolopoulou, 2025; European Commission et al., 2025). Students’ experiences suggest that while they recognize GAI’s potential, practical obstacles still impede seamless adoption.

These findings underscore the need for institutional frameworks that support AI literacy, ethical use, and research integrity (UNESCO, 2023; Umanets et al., 2024). Addressing these barriers will be essential for enabling postgraduate students to fully leverage GAI as a tool for cognitive and research talent development.

Taken together, the six dimensions reveal a central pattern: students are open to using GAI for functional support tasks such as summarization, translation, and clarity enhancement, yet they remain critically cautious when it comes to validity, ethics, and scholarly judgment. This balance underscores the dual nature of GAI adoption—enthusiastic experimentation tempered by reflective oversight.

### 10.2. Discussion of the Second Research Question: Do Notable Variations Exist in Postgraduate Students’ Utilization of GAI for Enhancing Cognitive and Research Talent When Considered Across Gender, Age, Academic Department, Program Level, Academic Standing, Specialization, Degree of Technology Adoption, and Intensity of GAI Engagement?

#### 10.2.1. Gender

The findings show statistically significant gender-related variations in the utilization of GAI, with male postgraduate students reporting higher engagement across several dimensions. This pattern aligns with research documenting gender disparities in AI adoption, digital readiness, and technological confidence. Prior studies indicate that male students often report greater familiarity with AI-enabled tools (Al-Samarraie et al., 2025) and higher levels of digital literacy (Møgelvang et al., 2024; Maxwell et al., 2025). Donaldson (2025) similarly found that male learners were more likely to use AI for research-related tasks such as data analysis and academic writing.

These differences may reflect broader sociocultural patterns in technology adoption, where male learners tend to exhibit higher self-efficacy and perceived competence in digital environments. Variations in prior exposure, disciplinary norms, and confidence in evaluating AI-generated outputs may also contribute to these disparities (Long & Magerko, 2020; Zawacki-Richter et al., 2019). Studies further suggest that male students may be more inclined to experiment with emerging technologies despite uncertainties regarding accuracy or ethical implications (Suchanek & Kralova, 2025; Xiao et al., 2025).

Importantly, these differences do not imply inherent disparities in ability. Rather, they highlight the need for equitable AI training initiatives that address confidence gaps and ensure that all students—regardless of gender—can benefit from GAI. This aligns with UNESCO’s (2023) call for inclusive AI integration and with institutional recommendations emphasizing targeted support to reduce gender-based disparities (European Commission et al., 2025; Tlili et al., 2025).

#### 10.2.2. Age

The ANOVA findings reveal that there are no statistically significant variations in the utilization of GAI use across different age groups (F = 2.66, *p* = 0.072). This suggests that postgraduate students, regardless of age, engage with GAI at broadly comparable levels. Although previous studies have reported that older students may demonstrate more intentional or reflective technology use (Maxwell et al., 2025; Xiao et al., 2025), the current findings do not show age-based disparities in actual GAI adoption.

The absence of significant differences may reflect the widespread availability and normalization of AI tools across postgraduate education, enabling students of different ages to access and use GAI similarly. It may also indicate that age-related variations in digital readiness or metacognitive awareness (Sprengers, 2025; Zhong & Rosli, 2025; Suchanek & Kralova, 2025) do not translate into measurable differences in GAI use.

Overall, the findings suggest that age is not a determining factor in GAI adoption within this sample, underscoring the importance of providing AI literacy opportunities that support learners across all age groups (UNESCO, 2023; European Commission et al., 2025).

#### 10.2.3. Academic Department

The ANOVA findings reveal that there are no statistically significant variations in the utilization of GAI use across academic departments (F = 0.589, *p* = 0.556). This indicates that students from different disciplinary backgrounds—whether computational, applied, or humanities-oriented—use GAI at similar levels.

Although prior research has documented disciplinary differences in technology adoption and digital readiness (Zawacki-Richter et al., 2019; Suchanek & Kralova, 2025), the present findings suggest that GAI has become sufficiently accessible and widely integrated across academic contexts to minimize such disparities. This may reflect increasing cross-disciplinary exposure to AI tools, institutional efforts to promote digital transformation, or the growing relevance of GAI to diverse research tasks.

The lack of significant departmental differences highlights the broad applicability of GAI across fields and reinforces the need for institution-wide AI literacy initiatives that support students regardless of disciplinary affiliation (Nazyrova et al., 2025; Alshamsi et al., 2024; Mbah et al., 2025; Tlili et al., 2025).

#### 10.2.4. Program Level

No statistically meaningful variations were found between master’s and doctoral learners, suggesting that program type alone does not determine GAI use. Instead, factors such as academic experience, research stage, and digital readiness appear to play more influential roles (Zhong & Rosli, 2025; Suchanek & Kralova, 2025).

The absence of program-level differences may reflect the growing normalization of GAI across postgraduate education. As AI tools become more accessible, both master’s and doctoral students may be adopting them at similar rates (Alshamsi et al., 2024; Nazyrova et al., 2025). Both groups also face similar research challenges—such as synthesizing literature and refining academic writing—which GAI can support (Xiao et al., 2025; Sushereba et al., 2025).

These findings underscore the importance of providing unified AI training initiatives that serve students across all postgraduate pathways, consistent with UNESCO (2023) and European Commission et al. (2025) recommendations.

#### 10.2.5. Academic Level

Significant differences were observed based on academic level, with students at more advanced stages—particularly those preparing theses or dissertations—reporting higher levels of GAI use. This aligns with research showing that AI adoption increases as students encounter more complex research demands (Xiao et al., 2025; Sushereba et al., 2025).

Advanced students typically face greater pressure to synthesize literature, design rigorous studies, and produce publication-ready writing, which may explain their heightened reliance on GAI. Academic progression is also associated with increased digital readiness and stronger metacognitive awareness (Zhong & Rosli, 2025; Suchanek & Kralova, 2025).

These findings highlight the importance of introducing structured AI training early in postgraduate education to prevent widening gaps in research readiness. UNESCO (2023) and the European Commission et al. (2025) emphasize the need for equitable access to AI literacy across all academic stages.

#### 10.2.6. Specialization

The ANOVA findings further reveal no statistically meaningful variations in GAI use across program specializations (F = 0.872, *p* = 0.561). This indicates that students specializing in empirical, computational, or data-driven fields do not differ significantly from those in theoretical or qualitative disciplines in their overall use of GAI.

While earlier studies have suggested that students in STEM or analytically oriented programs may be more inclined to adopt AI tools (Umanets et al., 2024; Sobolenko et al., 2024; Moongela et al., 2025), the current findings imply that GAI adoption has become more evenly distributed across specializations. This may be due to the increasing relevance of GAI to a wide range of academic tasks—including writing, summarization, and idea generation—which transcend disciplinary boundaries.

The absence of specialization-based differences underscores the importance of ensuring equitable access to AI training and resources across all academic programs (Nazyrova et al., 2025; Alshamsi et al., 2024; UNESCO, 2023). It also suggests that GAI is emerging as a broadly applicable tool that supports research development across diverse fields.

#### 10.2.7. Level of Technology Use

Significant differences were observed based on students’ overall level of technology use. Students with higher digital proficiency reported substantially greater GAI use across all dimensions. This aligns with research showing that digital literacy and technological self-efficacy strongly predict adoption of emerging technologies (Maxwell et al., 2025; Møgelvang et al., 2024; Zawacki-Richter et al., 2019).

Students with stronger digital backgrounds may be more confident in navigating AI interfaces and evaluating AI-generated outputs (Xiao et al., 2025; Sobolenko et al., 2024). Conversely, students with lower digital readiness may face barriers such as limited confidence or difficulty assessing AI accuracy (Mbah et al., 2025; UNESCO, 2023).

These findings highlight the importance of integrating digital skills training into postgraduate curricula to ensure equitable access to AI-enhanced research environments (European Commission et al., 2025; Tlili et al., 2025).

#### 10.2.8. Level of GAI Use

Finally, significant differences were found based on students’ self-reported level of GAI use. Students with higher prior engagement demonstrated substantially greater use across all dimensions, suggesting that familiarity builds confidence and encourages deeper integration of GAI into academic workflows. This aligns with research showing that repeated exposure enhances strategic use of AI tools (Xiao et al., 2025; Sushereba et al., 2025; Sobolenko et al., 2024).

These findings also reflect the developmental nature of AI adoption, where initial experimentation evolves into more sophisticated use as students gain experience (Suchanek & Kralova, 2025; Yang, 2025). Students with higher AI literacy are better equipped to evaluate outputs, recognize biases, and apply verification strategies (Long & Magerko, 2020; Tlili et al., 2025).

Conversely, students with lower GAI use may lack confidence or awareness of AI’s potential benefits (Mbah et al., 2025; UNESCO, 2023). These differences underscore the need for targeted training and capacity-building initiatives to ensure equitable access to AI-enhanced research practices (European Commission et al., 2025; Alshamsi et al., 2024).

These insights translate into clear implications for institutional practice: universities should strengthen structured AI training, establish transparent governance processes, and build supportive environments for responsible experimentation. Such measures will ensure that postgraduate students can leverage GAI effectively while safeguarding academic integrity and research quality.

## 11. Conclusions

This study fosters a comprehensive understanding of how postgraduate students engage with Generative Artificial Intelligence (GAI) as part of their cognitive and research development. The findings reveal a research environment in transition, where GAI is increasingly recognized as a valuable academic partner, yet its adoption remains selective and shaped by individual readiness, institutional support, and the nature of the research task. Students appear to be integrating GAI in ways that enhance efficiency, improve clarity, and support access to information, particularly in tasks related to literature review, academic writing, and early-stage data interpretation.

At the same time, the results show that students maintain clear boundaries regarding the types of tasks they are willing to delegate to AI. They continue to rely on human judgment for activities requiring deep conceptual reasoning, theoretical structuring, and methodological precision. This selective approach reflects a balanced understanding of both the strengths and limitations of GAI and demonstrates that students view AI as a complementary tool rather than a replacement for scholarly expertise.

A notable conclusion emerging from this study is the strong ethical awareness demonstrated by postgraduate students. They exhibit a high level of responsibility in verifying AI-generated content, cross-checking information, and adhering to academic integrity standards. This ethical orientation suggests that students are developing a mature and critical stance toward AI use, recognizing the importance of human oversight in maintaining the quality and credibility of academic work.

Despite these positive trends, this study identifies several challenges that may hinder the full integration of GAI into postgraduate research. The most prominent of these is the lack of structured training, which leaves many students uncertain about how to use AI tools effectively for advanced research tasks. Concerns about privacy, data protection, and the appropriate use of AI further complicate adoption, highlighting the need for clear institutional guidelines and supportive learning environments.

The inferential analyses underscore the importance of academic experience and digital proficiency in shaping GAI use. Students at more advanced academic levels and those with higher levels of technological readiness are more likely to integrate AI into their research practices, suggesting that both experience and digital literacy play critical roles in enabling effective AI adoption.

Overall, this study concludes that while GAI holds significant potential for enhancing cognitive and research talent, its impact depends on thoughtful, responsible, and well-supported integration. To fully realize the benefits of GAI, higher education institutions must invest in training, establish clear policies, and cultivate environments that empower students to use AI confidently, ethically, and creatively. The findings highlight the need for a balanced approach—one that embraces innovation while safeguarding the intellectual rigor and ethical standards that define postgraduate scholarship. Importantly, the integration of GAI should be framed not only as a technical enhancement but also as part of broader professionalisation efforts in postgraduate education, positioning AI literacy and ethical competence as core elements of academic and research development.

## 12. Research Importance and Implications

The importance of this study lies in its contribution to the growing scholarly interest in understanding how Generative Artificial Intelligence (GAI) can enhance the cognitive and research capabilities of postgraduate students. As AI-driven tools increasingly shape academic inquiry, creativity, and knowledge production, examining their role in strengthening higher-order thinking, analytical reasoning, and research design skills has become essential for advancing talent development in higher education. This study provides empirical insights into how GAI supports students in navigating complex research tasks—such as constructing theoretical frameworks, analyzing data, and generating innovative solutions—while also highlighting variations in usage across demographic, academic, and technological factors. By identifying current levels of engagement and areas for improvement, the research offers evidence-based guidance for educators, institutions, and policymakers seeking to foster cognitive growth, research excellence, and responsible AI integration within learning environments. Ultimately, the study contributes to a deeper understanding of how emerging AI technologies can empower learners, enrich academic performance, and promote more equitable and effective pathways for developing intellectual and research talent.

### 12.1. Implications for Theory

The study refines theoretical models of human–AI collaboration by showing that students use GAI selectively—employing it for information processing, linguistic refinement, and conceptual clarification, while reserving tasks requiring deep reasoning and methodological precision for human judgment. This pattern supports a nuanced view of AI as a complementary partner rather than a substitute for scholarly expertise.

### 12.2. Implications for Practice

The results highlight the need for structured training to build AI literacy and confidence. Students demonstrate ethical awareness and responsible use, yet their engagement remains moderate due to limited preparation. Embedding AI literacy into curricula as a distinct skill set, and treating it as part of professional competence development, can help students apply GAI effectively across literature review, writing, and data interpretation.

### 12.3. Implications for Policy

Concerns about privacy, integrity, and reliability emphasize the importance of clear institutional frameworks. Universities should establish governance policies that define acceptable AI use, safeguard data, and ensure transparency. Such policies should be aligned with broader institutional strategies for professionalization, ensuring that AI adoption contributes to competence development, ethical practice, and sustainable research environments.

## 13. Limitations and Future Directions

While the study offers valuable insights into how postgraduate students use GAI to enhance cognitive and research talent, several limitations should be considered when interpreting the findings. These limitations also point to promising avenues for future research.

### 13.1. Limitations

This study relies on self-reported data, which may be influenced by students’ perceptions, confidence levels, or familiarity with AI tools. The sample is limited to postgraduate students within a specific educational context, which may restrict the generalizability of the findings to other institutions or cultural settings. Additionally, while the multidimensional scale captures a broad range of GAI-related practices, it does not directly assess the quality, accuracy, or originality of AI-supported academic outputs. Finally, the cross-sectional design limits the ability to draw causal conclusions about how GAI use influences the development of cognitive and research talent over time.

### 13.2. Future Directions

Future research could build on these findings in several ways. Longitudinal studies would provide deeper insight into how sustained exposure to GAI shapes students’ research competencies, academic maturity, and ethical awareness. Experimental or mixed-methods designs could help evaluate the effectiveness of specific training interventions or pedagogical strategies aimed at improving AI literacy. Expanding the sample to include diverse institutions, disciplines, and cultural contexts would enhance the generalizability of the findings and reveal potential variations in adoption patterns. Future studies could also examine the quality and reliability of AI-assisted academic work, offering a more comprehensive understanding of how GAI contributes to scholarly development. Additionally, exploring faculty perspectives and institutional readiness would provide a more holistic view of the ecosystem required to support responsible and impactful AI integration in postgraduate education.

## 14. Recommendations

Based on the study’s findings, several strategic recommendations are proposed to guide institutions in fostering responsible and sustainable AI adoption in higher education. First, universities should design structured training programs that progress from basic digital literacy to advanced research applications, ensuring equitable access for students across academic levels. Clear institutional guidelines are also essential, particularly in areas such as transparency, citation practices, data security, and academic integrity. Faculty engagement plays a critical role, as supervisors and instructors can provide mentorship, feedback, and support to help students critically evaluate AI outputs. Integrating AI literacy into postgraduate curricula will further reduce disparities in technological proficiency and promote equal opportunities for all learners.

In addition, institutions should cultivate supportive environments by offering secure AI platforms, peer-learning opportunities, and innovation-friendly cultures that balance experimentation with scholarly rigor. Differentiated support tailored to gender, digital readiness, and academic level can help address disparities in AI use. Ethical infrastructure must also be strengthened through investment in secure, institution-hosted AI tools that comply with privacy and integrity standards, thereby reducing risks associated with external platforms. Finally, AI adoption should be translated into professionalized pathways, including standardized evaluation rubrics for AI-assisted work, clear ethics and integrity guidelines, and robust data protection protocols. Collectively, these measures will enable universities to build responsible, equitable, and sustainable AI-enhanced research environments.

## Figures and Tables

**Table 1:** Profile of the sample’s demographic and academic attributes.

Variables	Sub Variables	N	%
Gender	Male	115	53.74%
	Female	99	46.26%
Age	Under 30 years old	47	21.96%
	From 30 to 40 years old	83	38.79%
	Over 40 years old	84	39.25%
Academic department	Teaching and learning	109	50.93%
	Educational leadership and policy	89	41.59%
	Psychology	16	7.48%
Program level	Master’s	94	43.93%
	Doctoral	120	56.07%
Academic level	First level	56	26.17%
	Second level	11	5.14%
	Third level	66	30.84%
	Fourth level	18	8.41%
	Thesis Preparation Stage	63	29.44%
Specialization	Curriculum and General Teaching Methods	13	6.07%
	Mathematics Curriculum and Instruction	17	7.94%
	Science Curriculum and Instruction	51	23.83%
	Islamic Studies Curriculum and Instruction	12	5.61%
	Arabic Language Curriculum and Instruction	7	3.27%
	Foundations of Islamic Education	12	5.61%
	Educational Technology	9	4.21%
	Measurement and Evaluation	4	1.87%
	Educational Leadership	8	3.74%
	Counseling and Psychological Guidance	12	5.61%
	Educational Administration and Supervision	69	32.24%
Level of technology use	High	109	50.93%
	Moderate	105	49.07%
Level of GAI use	High	80	37.38%
	Moderate	100	46.73%
	Low	34	15.89%
	Total	214	100%

**Table 2:** Pearson correlation values for individual item scores in relation to their respective dimension totals and the overall scale total (*n* = 214).

Items Number	Correlation with Its Dimension	Correlation with the Overall Scale	Items Number	Correlation with Its Dimension	Correlation with the Overall Scale
1	0.856 **	0.766 **	25	0.822 **	0.717 **
2	0.898 **	0.787 **	26	0.863 **	0.802 **
3	0.903 **	0.794 **	27	0.927 **	0.850 **
4	0.855 **	0.802 **	28	0.849 **	0.825 **
5	0.874 **	0.793 **	29	0.838 **	0.753 **
6	0.790 **	0.759 **	30	0.926 **	0.845 **
7	0.801 **	0.719 **	31	0.889 **	0.803 **
8	0.880 **	0.808 **	32	0.909 **	0.791 **
9	0.894 **	0.811 **	33	0.841 **	0.650 **
10	0.925 **	0.836 **	34	0.684 **	0.691 **
11	0.925 **	0.860 **	35	0.905 **	0.568 **
12	0.945 **	0.826 **	36	0.919 **	0.558 **
13	0.926 **	0.834 **	37	0.933 **	0.596 **
14	0.917 **	0.837 **	38	0.921 **	0.584 **
15	0.917 **	0.870 **	39	0.917 **	0.586 **
16	0.907 **	0.868 **	40	0.899 **	0.563 **
17	0.855 **	0.743 **	41	0.741 **	0.408 **
18	0.930 **	0.821 **	42	0.795 **	0.312 **
19	0.941 **	0.855 **	43	0.795 **	0.227 **
20	0.923 **	0.829 **	44	0.664 **	0.374 **
21	0.923 **	0.820 **	45	0.821 **	0.429 **
22	0.935 **	0.832 **	46	0.787 **	0.365 **
23	0.889 **	0.824 **	47	0.812 **	0.378 **
24	0.908 **	0.867 **	48	0.849 **	0.405 **

** Correlation is significant at the 0.01 level (2-tailed).

**Table 3:** Pearson correlation matrix illustrating inter-dimension relationships and associations with the overall scale total (*n* = 214).

Dimensions	GAI-LRTF	GAI-RDPF	GAI-DAI	GAI-AWRR	GAI-ERU	GAI-CRD C	GAI-RCT Scale
GAI-LRTF	1	0.872 **	0.816 **	0.801 **	0.593 **	0.268 **	0.909 **
GAI-RDPF	0.872 **	1	0.846 **	0.884 **	0.517 **	0.237 **	0.917 **
GAI-DAI	0.816 **	0.846 **	1	0.860 **	0.465 **	0.290 **	0.903 **
GAI-AWRR	0.801 **	0.884 **	0.860 **	1	0.520 **	0.248 **	0.909 **
GAI-ERU	0.593 **	0.517 **	0.465 **	0.520 **	1	0.269 **	0.691 **
GAI-CRD C	0.268 **	0.237 **	0.290 **	0.248 **	0.269 **	1	0.462 **
GAI-RCT Scale	0.909 **	0.917 **	0.903 **	0.909 **	0.691 **	0.462 **	1

** Correlation is significant at the 0.01 level (2-tailed).

**Table 4:** Confirmatory Factor Analysis Fit Indices for the Six-Factor Model of the GAI-RCT Scale.

Fit Index	Value	Recommended Threshold	Interpretation
χ^2^	1922.6	—	Significant (df = 959, *p* < .001)
χ^2^/df	2.00	≤3.00	Acceptable fit
CFI	0.928	≥0.90	Good fit
NFI	0.867	≥0.90	Marginal fit
NNFI (TLI)	0.915	≥0.90	Good fit
SRMR	0.086	≤0.08	Slightly above threshold
RMSEA	0.069	≤0.08	Acceptable fit (90% CI = 0.064–0.073)

**Table 5:** Reliability statistics for the scale (*n* = 214).

No.	Dimensions	No. of Items	Cronbach’s Alpha (α)	The Guttman Split-Half Coefficients
1	GAI-Supported Cognitive Skills in Literature Review and Theoretical Framework Development	8	0.948 **	0.922 **
2	GAI-Enhanced Research Design and Problem-Formulation Skills	8	0.974 **	0.954 **
3	GAI-Assisted Data Analysis and Interpretation Skills	8	0.971 **	0.959 **
4	GAI-Driven Academic Writing and Research Refinement Skills	8	0.957 **	0.936 **
5	Ethical and Responsible Use of GAI in Research	8	0.954 **	0.946 **
6	Challenges in Employing GAI for Cognitive and Research Development	8	0.910 **	0.906 **
	Total scale	48	0.979 **	0.984 **

** Correlation is significant at the 0.01 level (2-tailed).

**Table 6:** Criteria for Performance Levels Derived from Mean Scores and Percentages.

No.	Mean Range	Percentage Range	Level
1	1–2.8	10–28%	Very Low
2	>2.8–4.6	>28–46%	Low
3	>4.6–6.4	>46–64%	Moderate
4	>6.4–8.2	>64–82%	High
5	>8.2–10	>82–100%	Very High

**Table 7:** Descriptive statistical results for the overall scale and its six dimensions (*n* = 214).

No	Dimension	Mean	SD	%	Level	Rank
1	GAI-Supported Cognitive Skills in Literature Review and Theoretical Framework Development	6.35	2.48	63.53%	Moderate	2
2	GAI-Enhanced Research Design and Problem-Formulation Skills	5.82	2.78	58.18%	Moderate	4
3	GAI-Assisted Data Analysis and Interpretation Skills	5.43	2.97	54.31%	Moderate	6
4	GAI-Driven Academic Writing and Research Refinement Skills	5.66	2.82	56.55%	Moderate	5
5	Ethical and Responsible Use of Generative GAI in Research	7.80	2.50	78.03%	High	1
6	Challenges in Employing Generative GAI for Cognitive and Research Development	6.02	2.40	60.17%	Moderate	3
	Overall	6.18	2.15	61.80%	Moderate	

**Table 8:** Descriptive statistical results for the GAI-supported cognitive skills in literature review and theoretical framework development (*n* = 214).

No	Item	Mean	SD	%	Level	Rank
1	I use AI tools to accurately identify the key concepts and terminology related to my research topic.	6.55	2.76	65.51%	High	2
2	I rely on AI to obtain well-crafted summaries of lengthy studies to deepen my understanding of the literature.	6.44	2.78	64.44%	High	3
3	I use AI to organize and delineate the body of literature most relevant to my research problem.	6.38	2.74	63.79%	Moderate	4
4	I employ AI to design an initial, coherent structure for the theoretical framework chapter.	5.82	2.93	58.22%	Moderate	8
5	I use AI to explain and interpret complex educational theories in ways that support the development of the theoretical framework.	6.28	2.94	62.76%	Moderate	6
6	I rely on AI to translate content from international studies to enrich my literature review.	6.81	3.00	68.08%	High	1
7	I use AI to suggest precise keywords that enhance my searches in academic databases.	6.32	3.01	63.22%	Moderate	5
8	I depend on AI to compare different theoretical perspectives and analyze their strengths and weaknesses.	6.22	3.01	62.24%	Moderate	7
	Overall	6.35	2.48	63.53%	Moderate	

**Table 9:** Descriptive statistical results for the GAI-enhanced research design and problem-formulation Skills (*n* = 214).

No	Item	Mean	SD	%	Level	Rank
1	I use AI to formulate accurate research objectives derived from the study problem.	5.67	3.00	56.73%	Moderate	7
2	I rely on AI to generate measurable and testable research questions and hypotheses.	5.62	3.02	56.21%	Moderate	8
3	I use AI to propose suitable items for questionnaires or measurement instruments.	5.90	3.06	59.02%	Moderate	2
4	I employ AI to generate interview or focus-group questions that support deeper inquiry.	5.87	2.99	58.74%	Moderate	3
5	I use AI to organize the domains and sections of the research instrument in a systematic manner.	5.84	2.95	58.41%	Moderate	4
6	I employ AI to create scenarios or hypothetical situations used in performance-based assessments.	5.74	3.07	57.38%	Moderate	6
7	I use AI to improve the clarity of research instrument items and ensure their appropriateness for participants.	6.14	2.98	61.36%	Moderate	1
8	I rely on AI to identify relationships among variables and construct a coherent research model.	5.76	3.12	57.57%	Moderate	5
	Overall	5.82	2.78	58.18%	Moderate	

**Table 10:** Descriptive statistical results for the GAI-assisted data analysis and interpretation skills (*n* = 214).

No	Item	Mean	SD	%	Level	Rank
1	I use AI to interpret statistical outputs accurately, including tests such as the *t*-test and ANOVA.	5.00	3.26	49.95%	Moderate	8
2	I rely on AI to organize and analyze qualitative data obtained from interviews.	5.08	3.28	50.84%	Moderate	7
3	I use AI to systematically link the study’s findings to its hypotheses.	5.36	3.20	53.55%	Moderate	6
4	I benefit from AI in explaining the statistical concepts used in my research.	5.67	3.19	56.73%	Moderate	2
5	I employ AI to identify recurring patterns and themes within textual data.	5.58	3.31	55.79%	Moderate	3
6	I use AI to generate suggestions for appropriate charts and tables to present the results.	5.49	3.23	54.86%	Moderate	5
7	I rely on AI to analyze the study’s findings and compare them with previous research.	5.70	3.32	56.96%	Moderate	1
8	I use AI to draft an initial version of the results and discussion chapter.	5.58	3.26	55.79%	Moderate	4
	Overall	5.43	2.97	54.31%	Moderate	

**Table 11:** Descriptive statistical results for the GAI-driven academic writing and research refinement Skills (*n* = 214).

No	Item	Mean	SD	%	Level	Rank
1	I rely on AI for linguistic, grammatical, and spelling proofreading of research texts.	5.54	3.31	55.37%	Moderate	6
2	I use AI to rephrase long sentences in a clearer and more academically appropriate manner.	6.00	3.21	59.95%	Moderate	2
3	I employ AI to generate practical, actionable recommendations based on my study’s findings.	5.65	3.20	56.54%	Moderate	4
4	I rely on AI to prepare abstracts in both Arabic and English.	6.02	3.17	60.19%	Moderate	1
5	I use AI to standardize citation and referencing styles, such as APA or others.	5.64	3.26	56.36%	Moderate	5
6	I rely on AI to enhance coherence between the chapters and sections of the thesis.	5.32	3.20	53.18%	Moderate	8
7	I employ AI to generate a precise and meaningful title that reflects the study’s content.	5.40	3.19	54.02%	Moderate	7
8	I use AI to improve the linguistic quality and academic depth of the written content.	5.68	3.22	56.82%	Moderate	3
	Overall	5.66	2.82	56.55%	Moderate	

**Table 12:** Descriptive statistical results for the ethical and responsible use of GAI in research (*n* = 214).

No	Item	Mean	SD	%	Level	Rank
1	I review and revise AI-generated content before incorporating it into the research.	7.47	3.04	74.67%	High	7
2	I commit to citing the original sources recommended by AI tools.	6.44	3.37	64.39%	High	8
3	I treat AI as a supportive tool that enhances my understanding, rather than a substitute for my scholarly effort.	8.10	2.75	80.98%	High	3
4	I ensure that the AI-generated content I use is original and consistent with principles of academic integrity.	8.10	2.78	81.03%	High	2
5	I make sure that AI-generated content is appropriate for the educational context of the university.	7.99	2.76	79.86%	High	6
6	I verify that my use of AI complies with academic regulations governing its application in scientific research.	8.07	2.78	80.65%	High	4
7	I dedicate time to checking the accuracy and reliability of data provided by AI tools.	8.02	2.81	80.19%	High	5
8	I recognize the possibility of errors or hallucinations in AI outputs and approach them with critical caution.	8.25	2.68	82.48%	High	1
	Overall	7.80	2.50	78.03%	High	

**Table 13:** Descriptive statistical results for the challenges in employing GAI for cognitive and research development (*n* = 214).

No	Item	Mean	SD	%	Level	Rank
1	I find it challenging to distinguish between accurate and unreliable content generated by generative AI.	5.22	3.16	52.24%	Moderate	7
2	I lack sufficient knowledge of how to effectively use generative AI tools in research processes.	5.01	3.13	50.14%	Moderate	8
3	I have concerns about the risk of plagiarism or academic dishonesty when using generative AI.	6.63	3.08	66.31%	High	3
4	My academic program does not provide adequate training on how to employ generative AI in scientific research.	7.10	2.91	70.98%	High	1
5	I face technical challenges when interacting with generative AI platforms or their advanced settings.	5.63	3.14	56.31%	Moderate	6
6	I am concerned about privacy and data protection when relying on generative AI tools.	6.78	3.02	67.80%	High	2
7	I find it difficult to integrate AI-generated outputs with traditional academic requirements in research.	5.64	3.04	56.45%	Moderate	5
8	I encounter challenges in evaluating the originality and quality of texts or analyses produced by generative AI.	6.11	2.99	61.12%	Moderate	4
	Overall	6.02	2.40	60.17%	Moderate	

**Table 14:** Results of Independent-Samples *t*-Tests Examining Differences in GAI Utilization by gender, program level, and technology-use level.

Variables	Sub Variables	N	Mean	SD	DF	t	Sig. (2-Tailed)	Effect Size (d)	Effect Size Interpretation
Gender	Male	115	312.25	104.91	212	2.417	0.017	0.33	Moderate
	Female	99	278.46	98.43					
Program level	Master’s	94	283.05	103.71	212	−1.711	0.088	0.17	Not significant
	Doctoral	120	307.25	101.83					
Level of technology use	High	109	337.88	94.08	212	6.518	0.000	0.89	Large
	Moderate	105	253.79	94.61					

**Table 15:** ANOVA findings on differences in GAI utilization by age, department, academic level, specialization, and GAI-use level.

Variables	Source of Variance	Sum of Squares	DF	Mean Square	F	Sig.
Age	Between Groups	55,738.87	2	27,869.44	2.66	0.072
	Within Groups	2,209,273.47	211	10,470.49		
	Total	2,265,012.34	213			
Academic department	Between Groups	12,578.37	2	6289.18	0.589	0.556
	Within Groups	2,252,433.97	211	10,675.04		
	Total	2,265,012.34	213			
Academic level	Between Groups	1,872,401.55	4	468,100.39	249.19	0.000
	Within Groups	392,610.80	209	1878.52		
	Total	2,265,012.34	213			
Program specialization	Between Groups	93,262.18	10	9326.22	0.872	0.561
	Within Groups	2,171,750.16	203	10,698.28		
	Total	2,265,012.34	213			
Level of GAI use	Between Groups	974,298.55	2	487,149.28	79.64	0.000
	Within Groups	1,290,713.79	211	6117.13		
	Total	2,265,012.34	213			

**Table 16:** Post hoc (LSD) analysis results on differences in postgraduate learners’ use of GAI for advancing SERTPs across academic level and levels of GAI use.

Variables	Sub Variables (I)	Sub Variables (J)	Mean Difference (I–J)	Std. Error	Sig.
Academic level	First level	Second level	−51.80682 *	14.29	0.000
		Third Level	−106.92803 *	7.87	0.000
		Fourth Level	−184.40278 *	11.74	0.000
		Thesis Preparation Stage	−241.49802 *	7.96	0.000
	Second level	First level	51.80682 *	14.29	0.000
		Third Level	−55.12121 *	14.12	0.000
		Fourth Level	−132.59596 *	16.59	0.000
		Thesis Preparation Stage	−189.69120 *	14.16	0.000
	Third Level	First level	106.92803 *	7.87	0.000
		Second level	55.12121 *	14.12	0.000
		Fourth Level	−77.47475 *	11.52	0.000
		Thesis Preparation Stage	−134.56999 *	7.63	0.000
	Fourth Level	First level	184.40278 *	11.74	0.000
		Second level	132.59596 *	16.59	0.000
		Third Level	77.47475 *	11.52	0.000
		Thesis Preparation Stage	−57.09524 *	11.58	0.000
	Thesis Preparation Stage	First level	241.49802 *	7.96	0.000
		Second level	189.69120 *	14.16	0.000
		Third Level	134.56999 *	7.63	0.000
		Fourth Level	57.09524 *	11.58	0.000
Levels of GAI use	High	Moderate	61.47000 *	11.73	0.000
		Low	202.06765 *	16.01	0.000
	Moderate	High	−61.47000 *	11.73	0.000
		Low	140.59765 *	15.53	0.000
	Low	High	−202.06765 *	16.01	0.000
		Moderate	−140.59765 *	15.53	0.000

* The mean difference is significant at the 0.05 level.

## Data Availability

The data supporting the findings of this study are available from the corresponding author upon reasonable request.

## References

[B1-jintelligence-14-00053] Abdelmagid A. S., Al-Mohaya A. Y., Ibrahim A. M., Teleb A. A. (2025). Generative AI technology and creativity in smart digital content production among university students. International Journal of Information and Education Technology.

[B2-jintelligence-14-00053] Aldossary A. S., Aljindi A. A., Alamri J. M. (2024). The role of generative AI in education: Perceptions of Saudi students. Contemporary Educational Technology.

[B3-jintelligence-14-00053] Al-Samarraie H., Sarsam S. M., Alzahrani A. I., Chatterjee A., Swinnerton B. J. (2025). Gender perceptions of generative AI in higher education. Journal of Applied Research in Higher Education.

[B4-jintelligence-14-00053] Alshamsi I., Sadriwala K. F., Alazzawi F. J. I., Shannaq B. (2024). Exploring the impact of generative AI technologies on education: Academic expert perspectives, trends, and implications for sustainable development goals. Journal of Infrastructure, Policy and Development.

[B5-jintelligence-14-00053] Amini M., Lee K. F., Yiqiu W., Ravindran L. (2025). Proposing a framework for ethical use of AI in academic writing based on a conceptual review: Implications for quality education. Interactive Learning Environments.

[B6-jintelligence-14-00053] Andersen J. P., Degn L., Fishberg R., Graversen E. K., Horbach S. P. J. M., Kalpazidou Schmidt E., Schneider J. W., Sørensen M. P. (2025). Generative artificial intelligence (GenAI) in the research process—A survey of researchers’ practices and perceptions. Technology in Society.

[B7-jintelligence-14-00053] Arpaci I., Al-Emran M., Al-Qaysi N., Al-Sharafi M. A. (2025). What drives the use of generative artificial intelligence to promote educational sustainability? Evidence from SEM–ANN approach. TechTrends.

[B8-jintelligence-14-00053] Baskara F. X. R. (2024). Generative AI as an enabler of sustainable education: Theoretical perspectives and future directions. British Journal of Teacher Education and Pedagogy.

[B9-jintelligence-14-00053] Benvenuti M., Cangelosi A., Weinberger A., Mazzoni E., Benassi M., Barbaresi M., Orsoni M. (2023). Artificial intelligence and human behavioral development: A perspective on new skills and competences acquisition for the educational context. Computers in Human Behavior.

[B10-jintelligence-14-00053] Castillo-Segura P., Fernández-Panadero C., Alario-Hoyos C., Kloos C. D. (2024). Enhancing research on engineering education: Empowering research skills through generative artificial intelligence for systematic literature reviews. 2024 IEEE Global Engineering Education Conference (EDUCON).

[B11-jintelligence-14-00053] Chen Y., Wang Y., Wüstenberg T., Kizilcec R. F., Fan Y., Li Y., Lu B., Yuan M., Zhang J., Zhang Z., Geldsetzer P., Chen S., Bärnighausen T. (2025). Effects of generative artificial intelligence on cognitive effort and task performance. Trials.

[B12-jintelligence-14-00053] Cheng A., Calhoun A., Reedy G. (2025). Artificial intelligence-assisted academic writing: Recommendations for ethical use. Advances in Simulation.

[B13-jintelligence-14-00053] Dinçer S. (2024). The use and ethical implications of artificial intelligence in scientific research and academic writing. Educational Research Implementation.

[B14-jintelligence-14-00053] Ding L., Lawson C., Shapira P. (2025). Rise of generative artificial intelligence in science. Scientometrics.

[B15-jintelligence-14-00053] Donaldson M. (2025). Do males use AI tools differently than female students when conducting social science research?. Ph.D. dissertation.

[B16-jintelligence-14-00053] Ersöz A. R., Engin M. (2024). Exploring ethical dilemmas in the use of artificial intelligence in academic writing: Perspectives of researchers. Journal of Uludag University Faculty of Education.

[B17-jintelligence-14-00053] Labrosse I., Campbell D., Karlstrøm H., Iversen E., Wang L., Notten A., European Commission (2025). The use of generative artificial intelligence in research.

[B18-jintelligence-14-00053] Giannakos M., Azevedo R., Brusilovsky P., Cukurova M., Dimitriadis Y., Hernández-Leo D., Järvelä S., Mavrikis M., Rienties B. (2025). The promise and challenges of generative AI in education. Behaviour & Information Technology.

[B19-jintelligence-14-00053] Jabli N. M., Al-Mohaya A. Y., Abdelmagid A. S., Ibrahim A. M. (2025). Exploring the impact of generative artificial intelligence on enhancing digital design thinking skills and academic creativity among university students. International Journal of Innovative Research in Science Studies.

[B20-jintelligence-14-00053] Kizilcec R. F., Huber E., Papanastasiou E. C., Cram A., Makridis C. A., Smolansky A., Zeivots S., Raduescu C. (2024). Perceived impact of generative AI on assessments. Computers and Education: Artificial Intelligence.

[B21-jintelligence-14-00053] Lee H.-P., Sarkar A., Tankelevitch L., Drosos I., Rintel S., Banks R., Wilson N. (2025). The impact of generative AI on critical thinking. CHI Conference on Human Factors in Computing Systems.

[B22-jintelligence-14-00053] Long D., Magerko B. (2020). What is AI literacy? Competencies and design considerations. Proceedings of the 2020 CHI conference on human factors in computing systems (CHI ’20).

[B23-jintelligence-14-00053] Malloy T., Gonzalez C. (2024). Applying generative artificial intelligence to cognitive models of decision making. Frontiers in Psychology.

[B24-jintelligence-14-00053] Maxwell D., Oyarzun B., Kim S., Bong J. Y. (2025). Generative AI in higher education: Demographic differences in student perceived readiness, benefits, and challenges. TechTrends.

[B25-jintelligence-14-00053] Mbah M. F., Nugraha T. R., Kushnir I. (2025). Challenges and opportunities for leveraging generative AI for sustainability education. Sustainability.

[B26-jintelligence-14-00053] Miao J., Thongprayoon C., Suppadungsuk S., Garcia Valencia O. A., Qureshi F., Cheungpasitporn W. (2024). Ethical dilemmas in using AI for academic writing. Clinical Practice.

[B27-jintelligence-14-00053] Moongela H., Matthee M., Turpin M., van der Merwe A. (2025). The effect of generative artificial intelligence on cognitive thinking skills in higher education institutions. Artificial Intelligence Research.

[B28-jintelligence-14-00053] Møgelvang A., Bjelland C., Grassini S., Ludvigsen K. (2024). Gender differences in the use of generative artificial intelligence chatbots in higher education. Education Sciences.

[B29-jintelligence-14-00053] Nazyrova A., Miłosz M., Bekmanova G., Omarbekova A., Aimicheva G., Kadyr Y. (2025). The digital transformation of higher education in the context of an AI-driven future. Sustainability.

[B30-jintelligence-14-00053] Nedungadi P., Tang K.-Y., Raman R. (2024). The transformative power of generative artificial intelligence for achieving the sustainable development goal of quality education. Sustainability.

[B31-jintelligence-14-00053] Nikolopoulou K. (2025). Generative artificial intelligence and sustainable higher education: Mapping the potential. Journal of Digital Educational Technology.

[B32-jintelligence-14-00053] Qi J., Xu Y., Liu J., Xue K. (2025). The impact of generative artificial intelligence tools on college students’ critical thinking and autonomous learning ability. Frontiers of Education in China.

[B33-jintelligence-14-00053] Shahzad M. F., Xu S., Zahid H. (2025). Exploring the impact of generative AI-based technologies on learning performance. Education and Information Technologies.

[B34-jintelligence-14-00053] Sobolenko L., Davydiuk A., Kornieva V., Lopatynska I., Bazyl O. (2024). Optimisation of learning and development of cognitive skills through virtual reality and artificial intelligence. E-Learning Innovation Journal.

[B35-jintelligence-14-00053] Sprengers J. O. (2025). The effect of age on generative AI usage, and the mediating role of technological stress. Bachelor’s thesis.

[B36-jintelligence-14-00053] Stein A. L. (2025). Generative AI and sustainability. The Oxford handbook of the foundations and regulation of generative AI.

[B37-jintelligence-14-00053] Suchanek P., Kralova M. (2025). Generative artificial intelligence expectations and experiences in management education. Journal of Innovation & Knowledge.

[B38-jintelligence-14-00053] Sushereba C. E., Militello L. G., Morris R., Ramachandran S., Diiulio J., Sarin A. (2025). Integrating generative artificial intelligence into interdisciplinary research. Proceedings of the Human Factors and Ergonomics Society Annual Meeting.

[B39-jintelligence-14-00053] Sweller J. (2020). Cognitive load theory and educational technology. Educational Technology Research and Development.

[B40-jintelligence-14-00053] Tlili A., Bond M., Bozkurt A., Arar K., Chiu T. K. F., Rospigliosi P. (2025). Academic integrity in the generative AI era. Interactive Learning Environments.

[B41-jintelligence-14-00053] Umanets V., Shakhina I., Rozputnia B. (2024). Training future computer science teachers to use artificial intelligence technologies. Modern Information Technologies and Innovative Methodologies in Education.

[B42-jintelligence-14-00053] UNESCO (2023). Guidance for generative AI in education and research.

[B43-jintelligence-14-00053] Vieriu A. M., Petrea G. (2025). The impact of artificial intelligence (AI) on students’ academic development. Education Sciences.

[B44-jintelligence-14-00053] Wang X., Zainuddin Z., Hai Leng C. (2025). Generative artificial intelligence in pedagogical practices: A systematic review of empirical studies (2022–2024). Cogent Education.

[B45-jintelligence-14-00053] Xiao L., Pyng H. S., Ayub A. F. M., Zhu Z., Gao J., Qing Z. (2025). University students’ usage of generative artificial intelligence for sustainability: A cross-sectional survey from China. Sustainability.

[B46-jintelligence-14-00053] Yang H. (2025). Harnessing generative AI: Exploring its impact on cognitive engagement, emotional engagement, learning retention, reward sensitivity, and motivation through reinforcement theory. Learning and Motivation.

[B47-jintelligence-14-00053] Zawacki-Richter O., Marín V. I., Bond M., Gouverneur F. (2019). Systematic review of research on artificial intelligence in higher education. International Journal of Educational Technology in Higher Education.

[B48-jintelligence-14-00053] Zhang Q., Han X. (2025). Research on cognitive engagement in graduate students’ human–computer interaction supported by generative AI. Proceedings of the 2025 2nd international symposium on artificial intelligence for education (ISAIE 2025).

[B49-jintelligence-14-00053] Zhao J., Chapman E., Sabet P. G. P. (2024). Generative AI and educational assessments: A systematic review. Education Research and Perspectives.

[B50-jintelligence-14-00053] Zhao Y., Yue Y., Sun Z., Jiang Q., Li G. (2025). Does generative artificial intelligence improve students’ higher-order thinking? A meta-analysis based on 29 experiments and quasi-experiments. Journal of Intelligence.

[B51-jintelligence-14-00053] Zhong Y., Rosli M. S. B. (2025). Generative artificial intelligence in higher education: Opportunities, challenges, and future directions. International Journal of Academic Research in Progressive Education and Development.

